# Reliability and validity of an agility‐like incremental exercise test with multidirectional change‐of‐direction movements in response to a visual stimulus

**DOI:** 10.14814/phy2.13275

**Published:** 2017-05-14

**Authors:** Dennis‐Peter Born, Philipp Kunz, Billy Sperlich

**Affiliations:** ^1^ Integrative and Experimental Exercise ScienceInstitute of Sport ScienceUniversity of WuerzburgWuerzburgGermany

**Keywords:** Change‐of‐direction movement, repeated sprint ability, speed, team sport, Yo‐Yo intermittent recovery

## Abstract

The aim of the study was to evaluate the reliability and validity of cardiorespiratory and metabolic variables, that is, peak oxygen uptake (*V*'O_2peak_) and heart rate (HR_peak_), obtained from an agility‐like incremental exercise test for team sport athletes. To investigate the test–retest reliability, 25 team sport athletes (age: 22 ± 3 years, body mass: 75 ± 7 kg, height: 182 ± 6 cm) performed an agility‐like incremental exercise test on the SpeedCourt (SC) system incorporating multidirectional change‐of‐direction (COD) movements twice. For each step of the incremental SC test, the athletes covered a 40‐m distance interspersed with a 10‐sec rest period. Each 40 m distance was split into short sprints (2.25–6.36 m) separated by multidirectional COD movements (0°–180°), which were performed in response to an external visual stimulus. All performance and physiological data were validated with variables obtained from a ramp‐like treadmill and Yo‐Yo intermittent recovery level 2 test (Yo‐Yo IR2). The incremental SC test revealed high test–retest reliability for the time to exhaustion (ICC = 0.85, typical error [TE] = 0.44, and CV% = 3.88), *V*'O_2peak_, HR_peak_, ventilation, and breathing frequency (ICC = 0.84, 0.72, 0.89, 0.77, respectively). The time to exhaustion (*r *=* *0.50, 0.74) of the incremental SC test as well as the peak values for *V*'O_2_ (*r *=* *0.59, 0.52), HR (*r *=* *0.75, 0.78), ventilation (*r *=* *0.57, 0.57), and breathing frequency (*r *=* *0.68, 0.68) were significantly correlated (*P *≤* *0.01) with the ramp‐like treadmill test and the Yo‐Yo IR2, respectively. The incremental SC test represents a reliable and valid method to assess peak values for *V*'O_2_ and HR with respect to the specific demand of team sport match play by incorporating multidirectional COD movements, decision making, and cognitive components.

## Introduction

Team sports match play involves repeated bouts of high‐intensity exercise followed by periods of incomplete recovery (Bradley et al. 2009; Bishop et al. 2011; Girard et al. 2011). The repeated sprint ability not only requires great anaerobic power in order to accelerate quickly and sprint fast, but also involves a well‐developed aerobic capacity, that is, peak oxygen uptake (*V*'O_2peak_), to provide optimal recovery and fatigue resistance during the successive sprints (Bishop et al. 2011; Girard et al. 2011). In order to evaluate the aerobic capacity and individualize the conditioning programs, the Yo‐Yo intermittent recovery level 2 test (Yo‐Yo IR2) is often used for the performance analysis of team sport athletes and investigate the player's *V*'O_2peak_ and peak heart rate (HR_peak_). The Yo‐Yo IR2 is an incremental exercise test that involves an intermittent running profile thereby simulating the repeated sprint character of team sports match play (Krustrup et al. 2006).

The Yo‐Yo IR2, however, involves a bidirectional movement pattern with consecutive 20 m sprints interspersed with predefined 180° change‐of‐direction (COD) movements, while the nature of team sports involves a much larger variety of COD movements (Bloomfield et al. 2007; Povoas et al. 2012; Karcher and Buchheit 2014) and usually much shorter sprint distances (Datson et al. 2016). During match play, the athletes perform frequent turns, twists, sidesteps, breaking actions, and COD movements with various angles (Bloomfield et al. 2007; Povoas et al. 2012; Karcher and Buchheit 2014). More importantly, most COD movements in team sport have an agility‐like character since they are performed in response to an external stimulus, that is, continuously changing game situations, tactical maneuvers, ball movement, and several interacting opponents (Sheppard and Young 2006; Bloomfield et al. 2007; Wagner et al. 2014). The frequent cognitive and decision‐making time component of the agility‐like movement pattern in addition to the multidirectional COD movements must result in a fairly different cardiorespiratory and metabolic response compared to the bilateral Yo‐Yo IR2.

To simulate the multidirectional COD movements in response to an external stimulus, the so‐called SpeedCourt (SC) has been developed recently. The SC is a platform with the dimensions of 5.25 × 5.25 m and 12 contact plates arranged in a symmetric order. A screen highlights a random order of contact plates. Successively, when a player's foot touchdown is registered on the targeted contact sensor, the next running path is randomly visualized to the player. Thereby, the player performs short sprints with multidirectional COD movements in response to an external stimulus, including quick acceleration and braking actions with movements backward and sideways.

Since the Yo‐Yo IR2 does not mirror the multidirectional and agility‐like movement pattern in team sport, the aim of the study was to investigate the test–retest reliability and validity of an incremental exercise test on the SC involving a random multidirectional running profile with agility‐like movement pattern. Our hypothesis was that the cardiorespiratory and metabolic variables, that is, the peak values for *V*'O_2_ and HR, derived from the novel incremental SC test are reliable in team sport athletes and valid when compared to the conventional procedures of performance analysis, that is, ramp‐like treadmill test and Yo‐Yo IR2.

## Material and Methods

### Participants

For the present study, we recruited 25 male recreational team sport athletes (age: 22.5 ± 3.4 years, body mass: 74.7 ± 7.1 kg, height: 1.82 ± 0.06 m) competing for at least 6 years on a regional level in soccer, handball, basketball, or field hockey with at least two training sessions (90–120 min) and one competitive match per week. The exclusion criteria for the participation in the study were the use of medication, smoking, any neuromuscular or cardiorespiratory injuries, or diseases. After being informed of the potential risks and benefits involved, all athletes provided their written consent to participate. The study design was in accordance with the Declaration of Helsinki and approved by the ethical review board of the University of Wuerzburg.

### Study design

**Figure 1:**
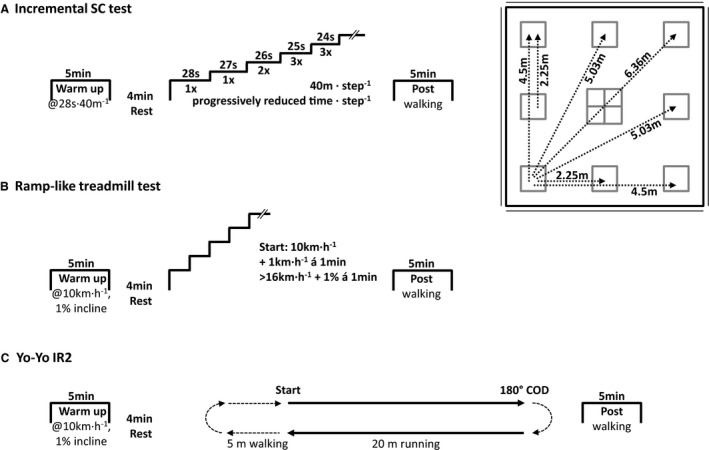
The protocols for the (A) incremental SpeedCourt (SC) test including randomized multidirectional COD movements in response to a visual stimulus, (B) ramp‐like treadmill test, and (C) Yo‐Yo intermittent recovery level 2 test (Yo‐Yo IR2) including predefined 180° COD movements. COD, change of direction.

The participants attended to all exercise tests well hydrated, wearing the same pair of shoes, and refrained from alcohol and strenuous exercise for 24 h as well as food for 3 h before testing. All tests, that is, the incremental SC test, ramp‐like treadmill test, and the Yo‐Yo IR2 (Fig. 1), were performed indoors to standardize the ambient temperature and air conditions. In addition, the tests were performed in a randomized order to minimize potential learning effects throughout the study.

After the familiarization trial, the test–retest reliability of the incremental SC test was investigated with all 25 participants. The performance, cardiorespiratory, and metabolic data were validated with a ramp‐like treadmill test and the Yo‐Yo IR2. In addition, 10 participants performed the ramp‐like treadmill test and Yo‐Yo IR2 twice to verify their reliability for the present laboratory setting.

During the tests the participants were equipped with a portable breath‐by‐breath gas analyzer (MetaMax3B_R2, Cortex Biophysik GmbH, Leipzig, Germany). Before, at the end, and 5 min after each test, the capillary blood lactate concentration was measured from the right ear lobe (LactatePro2, LT‐1730, Arkray, Kyoto, Japan).

### Ramp‐like treadmill test and Yo‐Yo IR2

The tests were initiated with a warm‐up at 10 km h^−1^ for 5 min followed by a 4‐min rest period. The ramp‐like treadmill test (H/P Cosmos, Mercury, Nussdorf‐Traunstein, Germany) started at 10 km h^−1^ (1% incline) and running velocity increased every 30 sec by 1 km h^−1^ as performed previously (Krustrup et al. 2006). To adapt the protocol for our recreational athletes, however, the running velocity was increased up to 16 km h^−1^. Thereafter, the running velocity was kept at 16 km h^−1^ with increasing inclination of 1% every 30 sec until voluntary exhaustion. In addition to the leveling off in *V*'O_2_, which was defined as an increase in *V*'O_2_ of <2.1 mL kg^−1^ min^−1^ (Taylor et al. 1955), maximal effort was considered when the athletes met two of the following three criteria: (1) respiratory exchange ratio (RER) ≥ 1.10, (2) blood lactate concentration ≥ 8 mmol L^−1^, and (3) perceived exertion was rated ≥ 18 on Borg's 6–20 scale (Borg 1970) as recommended earlier (Gore 2000). After the tests, the participants walked for 5 min at 3 km h^−1^. The Yo‐Yo IR2 was performed as described in detail elsewhere (Krustrup et al. 2006). Briefly, during the Yo‐Yo IR2 the athletes ran 2 × 20 m interspersed with a preplanned 180° COD movement and followed by 10 sec recovery period. The beginning, middle, and end of each sequence are marked with an acoustic signal and the running velocity continuously increased until voluntary exhaustion.

### Incremental SpeedCourt test

The incremental SC test was designed based on the sprint distances and progressively increasing running velocity of the Yo‐Yo IR2 (Krustrup et al. 2006). For each step of the incremental SC test, the participants had to cover a 40‐m distance on the SC followed by a 10‐sec rest period. For the first step, the running velocity was set at a moderate exercise intensity of 28 sec per 40 m running distance. Thereafter, the running velocity progressively increased throughout the test until voluntary exhaustion (see Table 1).

**Table 1:** The times to complete each steps 40 m distance and the total accumulated distance throughout the incremental SC test

Steps	Time (sec) to complete		Accumulated distance (m)
	20 m	40 m	
1	14	28	40
2	13.5	27	80
3	13	26	120
	13	26	160
4	12.5	25	200
	12.5	25	240
	12.5	25	280
5	12	24	320
	12	24	360
	12	24	400
6	11.5	23	440
	11.5	23	480
	11.5	23	520
7	11	22	560
	11	22	600
	11	22	640
8	10.5	21	680
	10.5	21	720
	10.5	21	760
9	10	20	800
	10	20	840
	10	20	880
10	9.5	19	920
	9.5	19	960
	9.5	19	1000
11	9	18	1040
	9	18	1080
	9	18	1120
12	8.5	17	1160
	8.5	17	1200
	8.5	17	1240
13	8	16	1280
	8	16	1320
	8	16	1360
14	7.5	15	1400
	7.5	15	1440
	7.5	15	1480
15	7	14	1520
	7	14	1560
	7	14	1600

To challenge the athlete's team sport specific speed and agility, the 40 m distance of each step was split into sprint sequences of 2.25–6.36 m, interspersed with COD movements of 0°, 45°, 90°, 117°, 135°, 143°, 153°, 162°, and 180°. With each foot touchdown on the targeted contact plate, the SC software successively highlighted the next running path on a large screen. Since the running paths were randomized and unknown to the players beforehand, all COD movements were performed in response to an external visual stimulus previously defined as agility (Sheppard and Young 2006). In addition to short sprints and explosive COD movements, the incremental SC test required the athletes to perform quick braking actions and included movements forward, backward, and sideways.

The running velocity for each step of the incremental test was controlled by an acoustic signal at the beginning, midsection, and end of each step. When the athlete failed to cover the full 40 m distance within the given time slot, the test leader gave a warning signal for the first and stopped the test with the second time of failure. As warm‐up, the athletes repeated the first step of the incremental SC test (40 m of randomized running paths in 28 sec interspersed by 10 sec recovery) for 5 min followed by a 4‐min rest period. After the test, the athletes walked a randomized sequence of contact plates for 5 min.

### Cardiorespiratory data

The portable gas analyzer was strapped to the participant's chest with a special harness during all tests. The total system weighed 1.3 kg, including the external battery pack. The athletes breathed through a turbine flowmeter which was attached to a proper fitting face mask covering the mouth and nose (7450 Series V2™ Mask, Hans Rudolph, Inc., Shawnee, KS). To assure the least possible disturbance of the usual movement pattern and to make the participants move freely, all cables were secured with straps and tape. Before all tests, the oxygen (O_2_) and carbon dioxide (CO_2_) sensors were 2‐point calibrated with the ambient air conditions (20.93% O_2_ and 0.03% CO_2_) as well as the anticipated expiratory gas compound using calibration gas containing 15% O_2_ and 5% CO_2_ (UN 1950 Aerosols, Cortex Biophysik GmbH, Leipzig, Germany). Flow volume of the turbine was calibrated using a 3‐L syringe (M9474‐C, Medikro Oy, Kuopio, Finland). A chest belt was connected to the gas analyzer via Bluetooth to collect the HR (H7, Polar Electro Oy, Kempele, Finland) in time alignment with the respiratory data. All data were collected breath by breath and for the subsequent analysis filtered with a moving average of 15 data points.

### Statistical analysis

All data are presented in mean values with standard deviations (SD). The normal distribution was confirmed using Shapiro–Wilk's test and an alpha level ≤ 0.05 considered statistically significant. To determine the reliability, the coefficient of variation (CV), intraclass correlation coefficient (ICC), and typical error (TE) with corresponding confidence limits at 90% were calculated using a spreadsheet provided by Hopkins (2015). A satisfactory relative reliability was assumed with an ICC ≥ 0.70 (Baumgartner and Chung 2001) accepting absolute reliability with a CV < 5% (Atkinson and Nevill 1998; Hopkins 2000). The minimal detectable change (MDC) with 95% confidence limit was calculated using the formula.

$\text{MDC}=\text{SEM}\times \sqrt{2\times 1.96}$

while the standard error of measurement (SEM) was calculated as

$\text{SEM}=\text{SD}\times \sqrt{1-r}$

with *r* being Pearson's correlation coefficient of trials 1 and 2 (Donoghue et al. 2009).

Pearson's product moment correlation coefficient was also used to determine the validity and verify the performance data from the incremental SC test with the ramp‐like treadmill test and the Yo‐Yo IR 2. The correlations were classified as small, medium, large, and very large with a coefficient >0.3, >0.5, >0.7, and >0.9, respectively (Hopkins, 2002). To further present the comparison between the intermittent SC test with the ramp‐like treadmill test and Yo‐Yo IR2 graphically, data were plotted according to Bland and Altman (1999). The statistical analysis was performed using Excel 2010 (Microsoft Corp., Redmond, WA) and SPSS Statistics 23.0 (IBM Corp., Armonk, NY).

## Results

**Figure 2:**
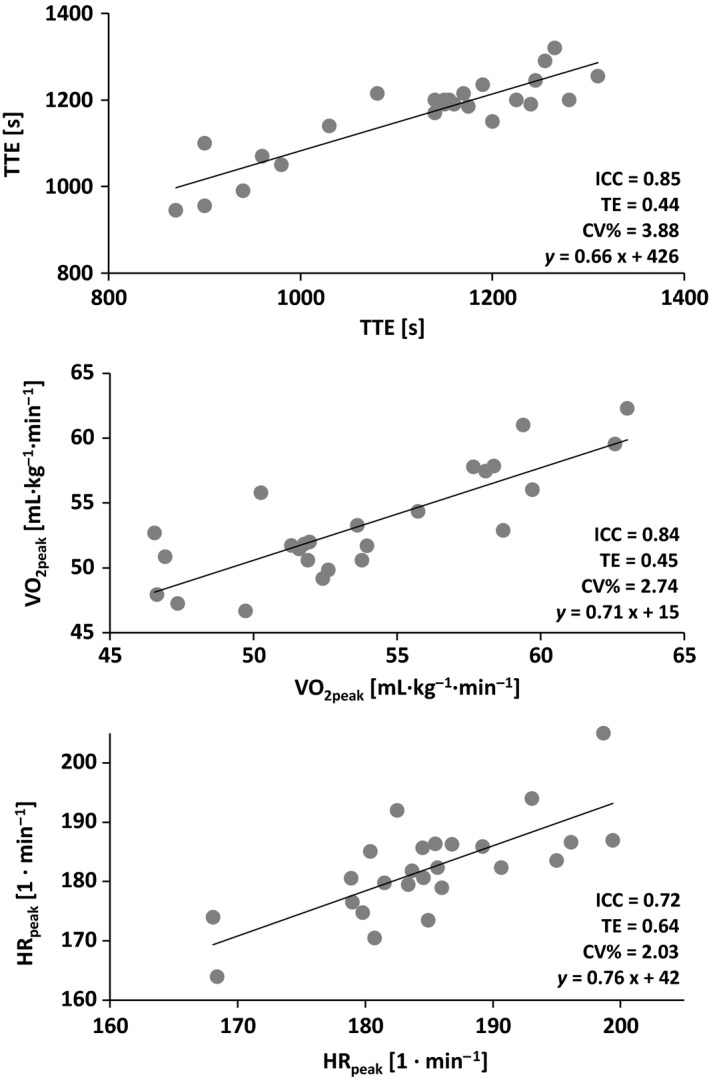
Test–retest reliability of the incremental SpeedCourt test with trial 1 on the *x*‐axis and trial 2 on the *y*‐axis. CV%, coefficient of variation; ICC, intraclass correlation coefficient; HR_peak_, peak heart rate; TE, typical error; TTE, time to exhaustion; *V*'O_2peak_, peak oxygen uptake.

The test–retest reliability for the incremental SC test is presented in Table 2 and Figure 2. The time to exhaustion (TTE) correlated largely between trial 1 and trial 2 (ICC = 0.85, TE = 0.44, CV% = 3.88). The *V*'O_2_, HR, ventilation, and breathing frequency correlated largely for all peak values (ICC ≥ 0.72, TE ≤ 0.64, and CV% ≤ 4.52) and 5 min post (ICC ≥ 0.78, TE ≤ 0.56, and CV% ≤ 9.63) between trial 1 and trial 2. The RER data 5 min post (ICC = 0.75, TE = 0.59, and CV% = 2.80) but not at peak (ICC = 0.57, TE = 0.90, and CV% = 3.73) correlated between trial 1 and trial 2. The blood lactate concentration and perceived exertion were poorly correlated for both, at peak and 5 min post (ICC ≤ 0.69, TE ≥ 0.70 and CV% ≥ 3.60) between trial 1 and trial 2.

**Table 2:** Mean values ± SD for the incremental SpeedCourt (SC) test and its test–retest reliability with intraclass correlation coefficient (ICC), typical error (TE), coefficient of variation (CV%), and minimal detectable change (MDC)

Parameter	Incremental SC test		ICC	TE	CV%	MDC
	Trial 1	Trial 2				
Peak values						
Time to exhaustion (min: sec)	18:44 ± 02:11	19:24 ± 01:36	0.85 (0.70–0.92)	0.44 (0.35–0.61)	3.88	75
Oxygen uptake (mL kg^−1^ min^−1^)	53.8 ± 4.8	53.3 ± 4.2	0.84 (0.69–0.92)	0.45 (0.36–0.62)	2.74	4
Heart rate (1 min^−1^)	185.1 ± 7.8	182.2 ± 8.2	0.72 (0.49–0.85)	0.64 (0.51–0.87)	2.03	8
Blood lactate concentration (mmol L^−1^)	6.2 ± 2.4	5.8 ± 2.1	0.69 (0.43–0.83)	0.70 (0.55–0.95)	16.03	3
Ventilation (L min^−1^)	137.1 ± 20.3	134.5 ± 17.7	0.89 (0.78–0.94)	0.36 (0.29–0.50)	4.52	14
Respiratory exchange ratio (a.u.)	1.08 ± 0.07	1.10 ± 0.05	0.57 (0.24–0.76)	0.90 (0.72–1.24)	3.73	0
Breathing frequency (1 min^−1^)	58.7 ± 6.7	58.1 ± 8.6	0.77 (0.57–0.88)	0.56 (0.45–0.77)	4.35	6
Ratings of perceived exertion (a.u.)	18.3 ± 1.1	18.2 ± 1.2	0.37 (−0.03–0.63)	1.34 (1.07–1.84)	3.60	2
5 min post						
Oxygen uptake (mL kg^−1^ min^−1^)	24.6 ± 6.0	22.3 ± 4.9	0.85 (0.71–0.92)	0.43 (0.34–0.60)	9.63	4
Heart rate (1 min^−1^)	135.3 ± 12.1	128.0 ± 9.1	0.78 (0.57–0.88)	0.56 (0.44–0.76)	4.48	9
Blood lactate concentration (mmol L^−1^)	5.4 ± 2.3	5.3 ± 2.1	0.51 (0.16–0.72)	1.01 (0.80–1.38)	20.04	3
Ventilation (L min^−1^)	55.6 ± 13.8	51.1 ± 11.8	0.88 (0.76–0.94)	0.38 (0.31–0.53)	8.96	8
Respiratory exchange ratio (a.u.)	0.87 ± 0.04	0.89 ± 0.06	0.75 (0.53–0.87)	0.59 (0.47–0.81)	2.80	0
Breathing frequency (1 min^−1^)	38.2 ± 7.0	36.6 ± 7.4	0.86 (0.72–0.92)	0.42 (0.34–0.58)	6.82	6
Ratings of perceived exertion (a.u.)	11.2 ± 2.2	11.6 ± 2.3	0.58 (0.26–0.77)	0.88 (0.70–1.20)	8.41	3

The ICC and TE are indicated with corresponding 90% confidence limits.

**Figure 3:**
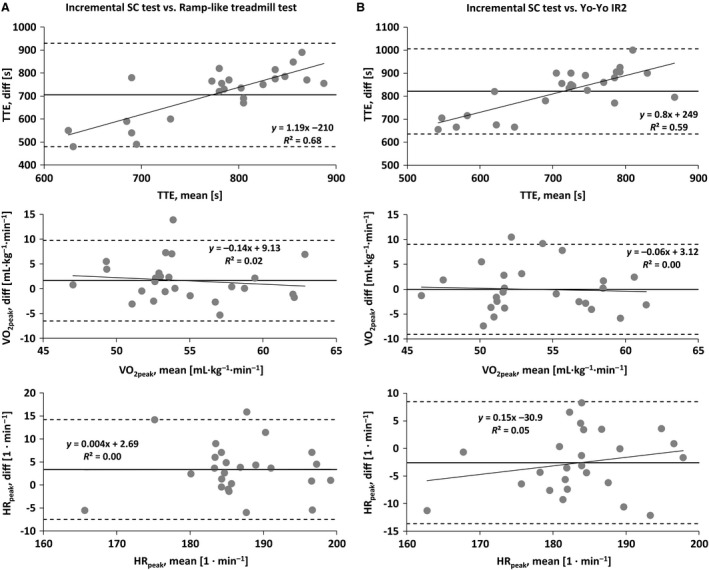
Bland–Altman plots for the comparison of the incremental SpeedCourt test with the (A) ramp‐like treadmill test and (B) Yo‐Yo intermittent recovery level 2 test (Yo‐Yo IR2). HR_peak_, peak heart rate; TTE, time to exhaustion; *V*'O_2peak_, peak oxygen uptake.

The TTE of the incremental SC test as well as the peak values for *V*'O_2_, HR, ventilation and breathing frequency correlated significantly with the ramp‐like treadmill test and the Yo‐Yo IR2 (*r* ≥ 0.50, *P* ≤ 0.01). At 5 min post, ventilation and breathing frequency correlated significantly with the ramp‐like treadmill test and Yo‐Yo IR2 (*r* ≥ 0.42, *P* ≤ 0.05) but not V'O_2_, HR, and perceived exertion (*r* ≤ 0.37, *P* > 0.05). The RER at 5 min post correlated with the Yo‐Yo IR2 (*r* = 0.62, *P* < 0.001), but not with RER of the ramp‐like treadmill test. Additionally, the TTE of the incremental SC test (*r* = 0.65, *P* < 0.01) as well as the Yo‐Yo IR2 (*r* = 0.75, *P* < 0.01) correlated closely with the *V*'O_2peak_ from the ramp‐like treadmill test. The detailed analysis of validity is presented in Table 3 and Figure 3.

**Table 3:** Analysis of validity for the incremental SpeedCourt (SC) test with the ramp‐like treadmill test and the Yo‐Yo intermittent recovery level 2 test (Yo‐Yo IR2) using Pearson's product–moment correlation coefficient

Incremental SC test	Ramp‐like treadmill test	*r*	Yo‐Yo IR2	*r*
Peak values				
Time to exhaustion (min: sec)	06:59 ± 00:46	0.50^b^	05:04 ± 01:02	0.74^c^
Oxygen uptake (mL kg^−1^ min^−1^)	55.5 ± 4.3	0.59^b^	53.8 ± 4.6	0.52^b^
Heart rate (1 min^−1^)	188.4 ± 7.8	0.75^c^	182.5 ± 9.0	0.78^c^
Blood lactate concentration (mmol L^−1^)	9.3 ± 3.9	0.30	9.2 ± 2.9	0.24
Ventilation (L min^−1^)	137.1 ± 18.6	0.57^b^	138.9 ± 20.3	0.57^b^
Respiratory exchange ratio (a.u.)	1.17 ± 0.05	0.19	1.22 ± 0.08	0.15
Breathing frequency (1 min^−1^)	50.5 ± 7.4	0.68^c^	51.9 ± 7.1	0.68^c^
Ratings of perceived exertion (a.u.)	18.9 ± 0.8	0.36	18.5 ± 1.1	0.29
5 min post				
Oxygen uptake (mL kg^−1 ^min^−1^)	15.2 ± 1.5	0.21	24.8 ± 5.8	0.35
Heart rate (1 min^−1^)	119.7 ± 12.6	0.24	130.4 ± 13.7	0.37
Blood lactate concentration (mmol L^−1^)	11.2 ± 3.8	0.46^a^	10.2 ± 3.6	0.32
Ventilation (L min^−1^)	46.2 ± 7.7	0.42^a^	65.6 ± 13.3	0.50^b^
Respiratory exchange ratio (a.u.)	1.06 ± 0.08	0.07	1.03 ± 0.09	0.62^c^
Breathing frequency (1 min^−1^)	30.8 ± 6.9	0.78^c^	33.8 ± 7.1	0.74^c^
Ratings of perceived exertion (a.u.)	12.2 ± 2.4	0.23	11.8 ± 2.2	0.30

Statistical significant correlations are indicated as follows:

a *P *≤* *0.05.

b *P *≤* *0.01.

c *P *≤* *0.001.

The test–retest reliability for the ramp‐like treadmill test was acceptable for the TTE (ICC = 0.85, TE = 0.49, CV% = 2.84), *V*'O_2peak_ (ICC = 0.69, TE = 0.76, CV% = 3.11), HR (ICC = 0.88, TE = 0.43, CV% = 1.64), blood lactate concentration (ICC = 0.45, TE = 1.27, CV% = 24.56), ventilation (ICC = 0.94, TE = 0.28, CV% = 2.51), and breathing frequency (ICC = 0.96, TE = 0.23, CV% = 3.43). Similarly, the Yo‐Yo IR2 showed an adequate test–retest reliability for the TTE (ICC = 0.85, TE = 0.49, CV% = 6.63), *V*'O_2peak_ (ICC = 0.73, TE = 0.68, CV% = 3.22), HR (ICC = 0.92, TE = 0.35, CV% = 1.54), blood lactate concentration (ICC = 0.82, TE = 0.53, CV% = 12.64), ventilation (ICC = 0.83, TE = 0.52, CV% = 5.38), and breathing frequency (ICC = 0.83, TE = 0.52, CV% = 6.93).

## Discussion

The aim of the study was to evaluate the reliability and validity of the cardiorespiratory and metabolic variables derived from the agility‐like incremental exercise test on the SC that incorporates multidirectional COD movements in response to an external visual stimulus. The main findings of the present investigation were that the incremental SC test showed an acceptable relative and absolute test–retest reliability for the TTE, *V*'O_2peak_, and HR_peak_, which are also valid measures when compared to the values obtained from a ramp‐like treadmill test and the Yo‐Yo IR2.

The Yo‐Yo IR2 showed a high retest reliability and even serves as a valid indicator for *V*'O_2peak_ measurement when compared to ramp‐like treadmill testing (Krustrup et al. 2006). The Yo‐Yo IR2, however, consists of straight sprints interspersed with preplanned 180° COD movements, while team sport match play involves a greater variety of movement patterns, such as COD movements with various angles, quick breaking actions, fast accelerations, as well as movements backward and sideways (Bloomfield et al. 2007; Karcher and Buchheit 2014). Interestingly, during soccer match play most COD movements (609 ± 193 out of 726 ± 203) are performed with angles between 0° and 90° rather than 180° (Bloomfield et al. 2007). Furthermore, the majority of COD movements are not preplanned, but performed in response to an external stimulus. The so‐called agility involves the cognitive and decision‐making time component (Sheppard and Young 2006), and must result in a different cardiorespiratory and metabolic response when compared to the Yo‐Yo IR2 involving linear sprinting and preplanned COD movements.

In general, for validity assessment, obtained data from the novel method are compared with a “gold standard.” For the validity of *V*'O_2peak_ and HR_peak_ measurements, ramp‐like treadmill tests are favored (Gore 2000). In the present study, the correlation between the test performance in the incremental SC test and the *V*'O_2peak_ from the ramp‐like treadmill test was *r* = 0.65 (*P* < 0.01), which is in line with the previous correlations (*r* = 0.56, *P* < 0.05) found between the Yo‐Yo IR2 and ramp‐like treadmill testing (Krustrup et al. 2006). In both cases, although the correlation was significant, the data show that players who possess a high *V*'O_2peak_ in the ramp‐like treadmill test do not necessarily perform well during the Yo‐Yo IR2 or the incremental SC test.

Since both, the ramp‐like treadmill test and the Yo‐Yo IR2, do not mirror the multidirectional running profile of team sport match play, we may question whether those two tests are suitable as “gold standard” to assess the cardiorespiratory and metabolic capacity of team sport athletes. Therefore, in the present investigation we aimed to adapt the physiological performance analysis within the incremental SC test to the typical characteristics of team sports. The players had to randomly perform a variety of short sprints between 2.25 m and 6.36 m, and multidirectional COD movements between 0° and 180° in response to a visual stimulus. Here, the incremental SC test revealed to be a reliable test to investigate the TTE, *V*'O_2peak_, and HR (ICC > 0.70) incorporating multidirectional COD movements in response to an external visual stimulus. In addition, the TTE of the incremental SC test as well as the Yo‐Yo IR2 correlated closely with the *V*'O_2peak_ from the ramp‐like treadmill test (*r* ≥ 0.65, *P* < 0.01). Since the Yo‐Yo IR2 was also employed to estimate the *V*'O_2peak_ (Krustrup et al. 2006), we may conclude that the incremental SC test may also be applied to estimate a player's *V*'O_2peak_.

During the incremental SC test, the TTE was substantially longer when compared with the Yo‐Yo IR2 (~ 19 min vs. ~5 min). The incremental SC tests protocol has been developed based on the distances (40 m) and rest periods (10 sec) of the Yo‐Yo IR2. With the repeated COD movements, however, there is a decision‐making component added to the running profile. The perception of and reaction to the visual stimulus increased the time to cover the full 40 m distance for each step of the incremental SC test explaining why the TTE during the incremental SC test was substantially longer.

While the V'O_2peak_ (53.6 mL kg^−1^ min^−1^ vs. 53.8 mL kg^−1^ min^−1^) was still similar, the incremental SC test revealed substantially lower levels of blood lactate concentration compared to the Yo‐Yo IR2 (6.0 vs. 9.2 mmol L^−1^, respectively). The shorter sprint distances (2.25 m to 6.36 m) in connection with the repeated COD movements made it impossible to reach as high sprint speeds as with the Yo‐Yo IR2 leading to less metabolic demand and increased neuromuscular strain (Buchheit and Laursen 2013). In fact, COD movements of various angles (45°, 90°, and 135°) implemented into sprints decreased the levels of blood lactate compared to linear sprinting (Buchheit et al. 2012). Most probably, the excessive eccentric contractions during deceleration in repeated COD movements explain the reduced levels of blood lactate despite similar *V*'O_2peak_. Thereby, the individual strategies to perform the COD movements (a quite harsh start–stop movement pattern with each COD movement vs. a smoother running style with less breaking forces and therefore less energy expenditure for the subsequent acceleration especially with COD having an obtuse angles) might have contributed to the quite large variability in blood lactate concentration. The latter mechanism might also explain the poor reliability that has been shown for the ratings of perceived exertion. Moreover, previous studies found greater variability of perceived exertion with increasing exercise intensity during incremental tests (Lamb et al. 1999). Therefore, the *V*O_2peak_ and HR_peak_ should be investigated as the primary parameters of interest when using the incremental SC test for the performance analysis of team sport athletes.

The validity analysis showed that the TTE of both, the incremental SC test and the Yo‐Yo IR2, correlated to a high extent (*r* = 0.74, *P* ≤ 0.001). However, the Bland–Altman analysis revealed that the players with a greater mean TTE performed better (i.e., greater TTE) in the incremental SC test compared to the Yo‐Yo IR2, despite similar *V*'O_2peak_. Several factors may account for the discrepancy why players may perform better in one test compared to the other. For instance, the pure COD speed mainly relies on technical skills (Brughelli et al. 2008) and leg strength and power relative to the body mass (Delaney et al. 2015). The incremental SC test, however, adds the visual stimulus hence the cognitive and decision‐making component to the COD movement. This requires an effective and fast perception of and very quick reaction to the given external stimulus prior to the actual COD movement (Sheppard and Young 2006). In this context, the cognitive component rather than the actual speed of movement improved performance during the agility task (Young and Rogers 2014). Since the cognitive component for decision making requires time without stimulating the cardiorespiratory system, the very short “breaks” to respond to the visual stimulus before each COD movement might explain why the validity analysis revealed that only 16 (of 30) correlations were statistically significant with only five coefficients showing *r* > 0.70. Therefore, from a practical perspective, not only training but also performance analysis in team sport athletes should involve the specific cognitive component of agility, that is, perception of and reaction to an external stimulus.

While multiple agility tests for team sport athletes have been published recently, most tests aim to investigate the neuromuscular performance rather than the cardiorespiratory and metabolic capacity. For instance, Hachana et al. (2014) modified the well‐established Illinois agility test and reduced the sprint distances to better display the COD speed rather than the linear sprint ability. Spasic et al. (2015) showed a high test–retest reliability (ICC = 0.85, CV = 3%) using a T‐like run. In the latter study, the handball players performed a quick COD movement by 90° after deciding to turn left or right in response to a visual stimulus. During match play, however, the athletes have to perform multiple COD movements in various directions while moving forward, backward, and sideways (Bloomfield et al. 2007). The incremental SC test provides the opportunity to investigate the cardiorespiratory and metabolic capacity underlying a team sport specific oscillating running profile with agility‐like and multidirectional movement pattern to further improve the physiological performance analysis for team sport athletes.

### Limitations of the study

The incremental SC test is reliable and valid for the assessment of *V*'O_2peak_ and HR_peak_ with respect to the multidirectional movement pattern of team sport match play but does not involve a universal movement pattern for all team sports that might also involve longer running distances than the ones performed in the incremental SC test. Also, the running profiles may vary according to the sport, gender, level of performance, and age. With an initial approach the present study evaluated the incremental SC test in a broad spectrum of team sport athletes (including players from soccer, handball, basketball, and field hockey) to investigate the potential mismatch between variables derived from the incremental SC and the traditional performance tests, that is, the ramp‐like treadmill and Yo‐Yo IR2. Future investigations will need to adjust the running pattern according to the specific team sport and performance level.

Earlier reliability studies used multiple repeated measure analyses to investigate how many repetitions of a test are required to achieve adequate reliability from a within‐ and between‐day perspective (Donath and Wolf 2015; Roth et al. 2017). Due to the all‐out nature as well as high neuromuscular and aerobic demand of the incremental SC test, we refrained from determining the within‐day variation. Future studies, however, will need to determine the between‐day variation and investigate whether more repetitions could further improve the test–retest reliability of the incremental SC test.

## Conclusions

The main findings of the study were that the TTE, *V*'O_2peak_, and HR_peak_ obtained from an agility‐like incremental exercise test with multidirectional COD movements in response to an external visual stimulus showed high relative and absolute test–retest reliability and validity when compared to the data obtained from a ramp‐like treadmill test and the Yo‐Yo IR2. To further adapt the physiological performance analysis to the specific demand of team sport match play by incorporating multidirectional COD movements, decision making, and cognitive components, we may recommend the incremental SC test as reliable and valid method to assess the *V*'O_2peak_ and HR_peak_.

## Conflict of Interest

The authors have no conflict of interest to declare. The study was financed by own institutional resources.
